# Integrated analysis of the critical region 5p15.3–p15.2 associated with cri-du-chat syndrome

**DOI:** 10.1590/1678-4685-GMB-2018-0173

**Published:** 2019-04-11

**Authors:** Thiago Corrêa, Bruno César Feltes, Mariluce Riegel

**Affiliations:** 1 Post-Graduate Program in Genetics and Molecular Biology, Universidade Federal do Rio Grande do Sul, Porto Alegre, RS, Brazil; 2 Institute of Informatics, Universidade Federal do Rio Grande do Sul, Porto Alegre, RS, Brazil; 3 Medical Genetics Service, Hospital de Clínicas de Porto Alegre, Porto Alegre, RS, Brazil

**Keywords:** Cri-du-Chat Syndrome, 5p– cytogenomics, integrative Analysis, PPI, systems biology

## Abstract

Cri-du-chat syndrome (CdCs) is one of the most common contiguous gene syndromes, with an incidence of 1:15,000 to 1:50,000 live births. To better understand the etiology of CdCs at the molecular level, we investigated theprotein–protein interaction (PPI) network within the critical chromosomal region 5p15.3–p15.2 associated with CdCs using systemsbiology. Data were extracted from cytogenomic findings from patients with CdCs. Based on clinical findings, molecular characterization of chromosomal rearrangements, and systems biology data, we explored possible genotype–phenotype correlations involving biological processes connected with CdCs candidate genes. We identified biological processes involving genes previously found to be associated with CdCs, such as *TERT*, *SLC6A3,* and *CTDNND2*, as well as novel candidate proteins with potential contributions to CdCs phenotypes, including CCT5, TPPP, MED10, ADCY2, MTRR, CEP72, NDUFS6, and MRPL36. Although further functional analyses of these proteins are required, we identified candidate proteins for the development of new multi-target genetic editing tools to study CdCs. Further research may confirm those that are directly involved in the development of CdCs phenotypes and improve our understanding of CdCs-associated molecular mechanisms.

## Introduction

Cri-du-chat syndrome (CdCs, OMIM 123450) is one of the most common contiguous gene syndromes, with an incidence of 1:15,000 to 1:50,000 live births (Niebuhr, 1978; Duarte *et al.*, 2004). Although 5p deletion is clinically and genetically well described, the phenotypic variability observed among patients with the deletion suggests that additional modifying factors, including genetic and environmental factors, may impact patients’ clinical manifestations (Nguyen *et al.*, 2015). The classic phenotype of CdCs encompasses a cat-like cry, facial dysmorphism, microcephaly, psychomotor delays, and intellectual disability (Overhauser *et al.*, 1994). However, the clinical spectrum and severity of the disease depend of the size of the deleted chromosomal region (Smith *et al.*, 2010). Around 80% of individuals with CdCs exhibit *de novo* terminal deletions, and 5% exhibit interstitial deletions, where the deletion is most commonly inherited (Mainardi, 2006). In this sense, approximately 10–15% of the deletions result from an unbalanced parental translocation (Mainardi, 2006), whereas complex genomic rearrangements, such as mosaicism, *de novo* translocation, or ring chromosomes, comprise fewer than 10% of cases (Perfumo *et al.*, 2000).

Previous studies looking for phenotype–genotype correlations through determination of deleted regions on 5p have described critical regions related to increased susceptibility for cat-like cry, speech delay, facial dimorphism, and intellectual disability (Overhauser *et al.*, 1994; Church *et al.*, 1997; Marinescu *et al.*, 1999; Mainardi *et al.*, 2001; Zhang *et al.*, 2005; Elmakky *et al.*, 2014). Although studies differ in the actual contribution of these critical regions to a particular phenotype, they allow that refinement of genes under hemizygous conditions may contribute to the pathogenesis of CdCs (Mainardi, 2006; Damasceno *et al.*, 2016). Candidate genes, such as *TERT*, *MARCH6*, *CTNND2*, and *SLC6A3,* are considered dose-sensitive or conditionally haploinsufficient (i.e., a single copy of these genes is insufficient to ensure normal functioning in individuals with CdCs) (Nguyen *et al.*, 2015). Haploinsufficiency of the genes mentioned above has been implicated in telomere maintenance dysfunction, cat-like cry, intellectual disability, and attention-deficit/hyperactivity disorder, respectively (Wu *et al.*, 2005; Du *et al.*, 2007; Hofmeister *et al.*, 2015; Tong *et al.*, 2015).

Even with the increasing resolution of cytogenetic techniques and the large amount of information available in databases, the investigation of contiguous gene syndromes remains a challenge. Studies have attempted to characterize genomic rearrangements and establish genotype–phenotype correlations through the identification of critical regions of susceptibility to CdCs, candidate genes, and haploinsufficiency-related altered mechanisms implicated in CdCs phenotypes (Lupski and Stankiewicz, 2005; Nguyen *et al.*, 2015). Therefore, in this study, to better understand the etiology of CdCs at the molecular level, we applied an integrative approach that combines conventional cytogenetic techniques, chromosomal microarray analysis (CMA), and systems biology tools to elucidate the probable molecular mechanisms underlying the clinical conditions present in CdCs.

## Subjects and Methods

### Study design and sample selection

This is a retrospective cytogenomic integrative analysis involving results of a series of cases. Clinical and cytogenomic data were extracted from six patients with CdCs enrolled in the Brazilian Network of Reference and Information in Microdeletion Syndromes (RedeBRIM) project (Riegel *et al.*, 2014, 2017; De Souza *et al.*, 2015; Dorfman *et al.*, 2015). The patients were regularly reevaluated over several years. Psychomotor development assessments were based on personal observations, school performance, and parent information. Daily abilities and skills, such as language, social interactions, concentration/attention, impulsiveness, motor control, perception, and learning and memory were recorded and published by our group elsewhere (Damasceno *et al.*, 2016). The five most frequent groups of clinical findings were selected and registered in the present study. This study has been approved by the Ethics Research Committee of Hospital de Clínicas de Porto Alegre (HCPA), followed the Declaration of Helsinki, and the standards established by the author’s Institutional Review Board.

### Cytogenomic Small Region of Overlap (SRO)

The deletions were mapped by whole genome array-CGH using a 60-mer oligonucleotide-based microarray with a theoretical resolution of 40 kb (8 60K, Agilent Technologies Inc., Santa Clara, CA). Labeling and hybridization were performed following the protocols provided by Agilent 2011. The arrays were analyzed using a microarray scanner (G2600D) and Feature Extraction software (version 9.5.1) (both from Agilent Technologies). Image analyses were performed using Agilent GenomicWorkbench Lite Edition 6.5.0.18 with the statistical algorithm ADM-2 at a sensitivity threshold of 6.0. The detailed cytogenomic profiles of the patients analyzed in this study were presented by our group elsewhere (Damasceno *et al.*, 2016). Based on it, the chromosomal SRO was determined.

### Network design

The protein–protein interaction (PPI) metasearch engine STRING 10.0 (http://string-db.org/) was used to create PPI networks based on genes located in the SRO. The list of genes was obtained from the human assembly of February 2009 (GRCh37/hg19) (Kent *et al.*, 1976; von Mering *et al.*, 2005). The parameters used in STRING were: (i) degree of confidence, 0.400, with 1.0 being the highest level of confidence; (ii) 500 proteins in the 1^st^ and 2^nd^ shell; and (iii) all prediction methods enabled, except for text mining and gene fusion. The final PPI network obtained through STRING was analyzed using Cytoscape 3.5 (Shannon *et al.*, 2003). Non-connected nodes from the networks were not included.

### Clustering and GO analysis

The MCODE tool was used to identify densely connected regions in the final Cytoscape network. The analysis was based on vertex weighting by the local neighborhood density and outward traversal from a locally dense seed protein to isolate the highly clustered regions (Bader and Hogue 2003). The PPI modules generated by MCODE were further studied by focusing on major biology-associated processes using the Biological Network Gene Ontology (BiNGO) 3.0.3 Cytoscape plugin (Maere *et al.*, 2005). The degree of functional enrichment for a given cluster and category was quantitatively assessed *(p*-value) using a hypergeometric distribution. Multiple test correction was also implemented by applying the false discovery rate (FDR) algorithm (Benjamini and Hochberg 1995) at a significance level of *p* < 0.05.

### Centralities

Two major parameters of network centralities (node degree and betweenness) were used to identify H-B nodes from the PPI network using the Cytoscape plugin CentiScaPe 3.2.1 (Scardoni *et al.*, 2009). The node degree centrality indicates the total number of adjacent nodes that are connected to a unique node. Nodes with a high node degree are called hubs and have central functions in a biological network (Scardoni *et al.*, 2009). Furthermore, we also analyzed the betweenness score, which corresponds to the number of shortest paths between two nodes that pass through a node of interest. Thus, nodes with high betweenness scores, compared to the average betweenness score of the network, are responsible for controlling the flow of information through the network topology (Newman, 2006; Scardoni *et al.*, 2009). These nodes are called bottlenecks and are normally related to the control of information between groups of proteins (Scardoni *et al.*, 2009).

### Availability of data and materials

All data generated or analyzed during this study are included in this published article and its supplementary information files (Tables S1-S17).

## Results

**Figure 1:**
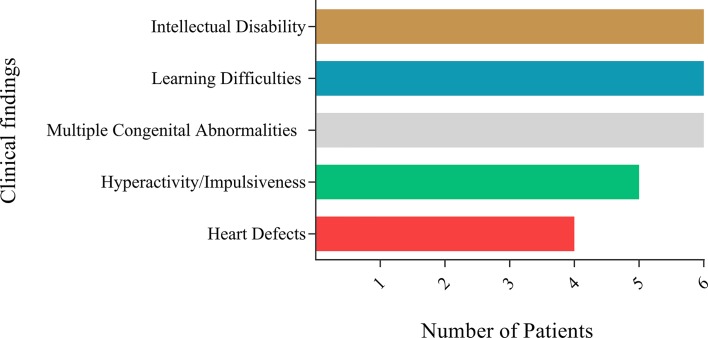
Summary of clinical findings of the six individuals in the study according to Damasceno *et al.* (2016).

The main clinical findings of six patients with CdCs selected to this study are presented in Figure 1. Intellectual disability (6/6 patients), learning difficulties (6/6 patients), multiple congenital abnormalities (6/6 patients), hyperactivity/impulsiveness (5/6 patients), and heart defects (4/6 patients) were the most frequent findings (Figure 1). Among the samples, three were from males, with ages ranging from 6 to 38 years, and three were from females, with ages ranging from 7 to 20 years.

### Cytogenomic data analysis MR

**Figure 2:**
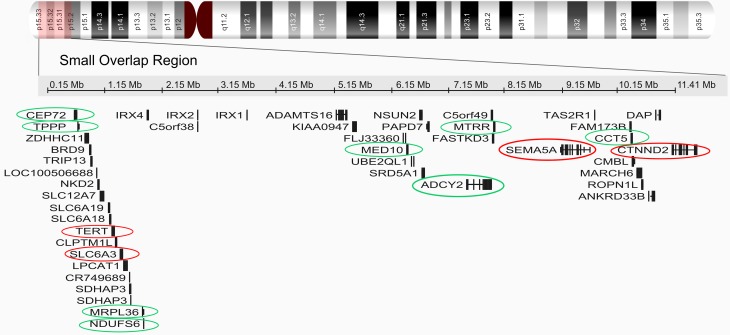
Cytogenomic profile of chromosome 5. Chromosomal critical region of 5p15.33–p15.2. Genes localized to the critical region were obtained from the human assembly of February 2009 (GRCh37/hg19). Red circles show genes already associated with CdCs. Green circles show candidate genes from this study for contributing to the phenotype in CdCs.

Six *de novo* terminal deletions that ranged in size from approximately 11.2 Mb to 28.6 Mb, with breakpoints from 5p15.2 to 5p13 were mapped. The analysis of CMA profile data revealed a small region of overlap (SRO) of 10.8 Mb encompassing the bands 5p15.33–p15.2. The approximate genomic position of the SRO is chr5:527552–11411700, comprising 44 genes according to the UCSC genome browser human assembly of February 2009 (GRCh38/hg19) (Figure 2).

### Networks and topological analysis

**Figure 3:**
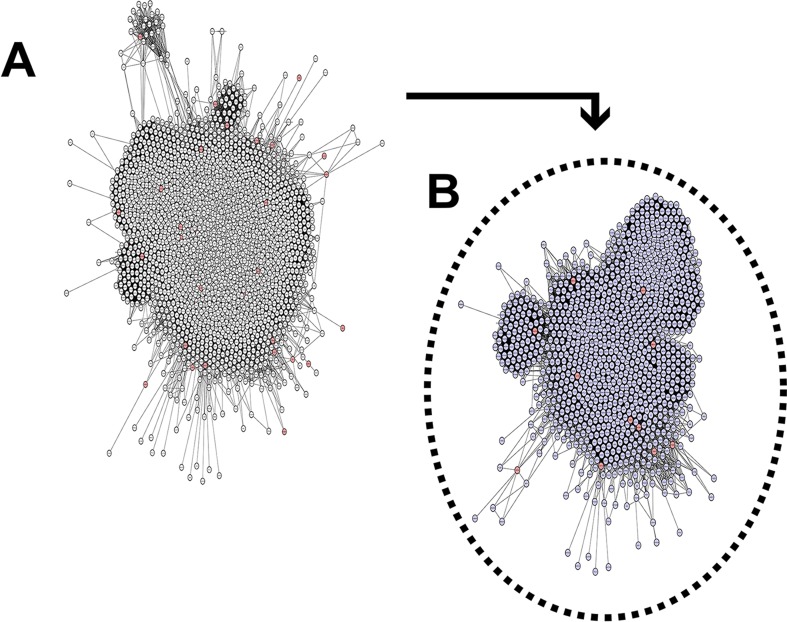
The PPI network. The list of 44 genes was obtained from the human assembly of February 2009 (GRCh37/hg19). Interaction data from STRING were used to construct networks using Cytoscape software. (A) The primary network is composed of 2284 nodes and 83,340 edges. Red nodes are target proteins (SLC6A3, SRD5A1, CCT5, ADCY2, TAS2R1, MED10, MTRR, SLC12A7, CEP72, NDUFS6, MARCH6, LPCAT1, NKD2, CTNND2, TERT, CLPTM1L, MRPL18, MRPL36, UBE2QL1, PAPD7, and TPPP). (B) Secondary network composed of 1062 nodes and 41,309 edges. Red nodes are candidate proteins (CCT5, TPPP, MED10, ADCY2, MTRR, CEP72, NDUFS6, MRPL36, CTNND2, TERT, and SLC6A3) and immediate neighbors from SRO.

Overall, the scale-free network was composed of 2284 nodes (proteins) and 83340 edges (interactions) (Figure 3). Centrality analyses were carried out to identify hub-bottlenecks (H-B), the most topologically relevant nodes. The network hubs (nodes with an above average number of connections) and betweenness (total number of non-redundant shortest paths going through a node or edge) indicate the most critical points in a biological network (Yu *et al.*, 2007). In our analysis, we observed 273 H-B nodes in the SRO network. Furthermore, we performed a cluster analysis that identified 16 major cluster regions above our cutoff score, and gene ontology (GO) analyses were performed in the identified modules.

**Figure 4:**
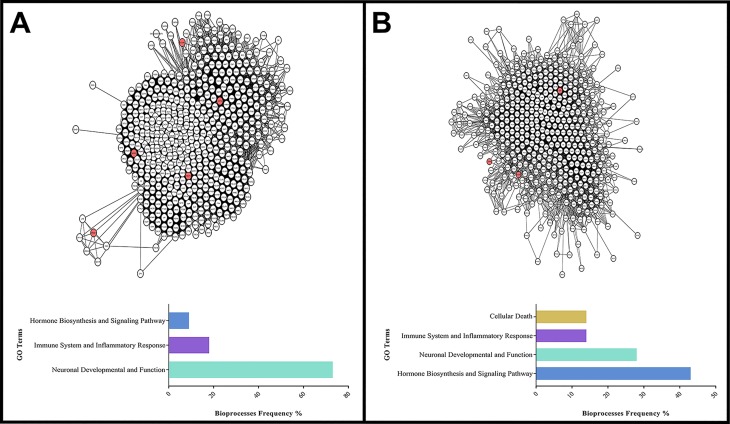
Subnetworks derived from clustering analysis. Red nodes are target proteins. (A) Cluster 1, with Ci = 94,369, composed of 509 nodes and 24,064 edges. Target proteins: SLC6A3, SRD5A1, CCT5, ADCY2, and TAS2R1. Below, summary of the bioprocess frequency identified in the PPI network (B) Cluster 8, Ci = 23,208, contains 471 nodes and 5477 edges. Target proteins: SLC6A3, TERT, and TPPP. Below, summary of the Bioprocess frequency identified in the PPI network.

Clusters taken into consideration for further analysis were those containing major proteins related to CdCs and deleted in all patients according to Espitiro Santo (2016), namely those containing combinations of SLC6A3, SRD5A1, CCT5, ADCY2, TAS2R1, MED10, MTRR, SLC12A7, CEP72, NDUFS6, MARCH6, LPCAT1, NKD2, CTNND2, TERT, CLPTM1L, MRPL18, MRPL36, UBE2QL1, PAPD7, and TPPP (Figure 4). In addition, the TERT protein was a commonly clusterized protein, and all clusters containing TERT were selected. Clusters that did not contain multiple combinations of the CdCs protein targets mentioned above, TERT, were excluded from the final analysis.

**Figure 5:**
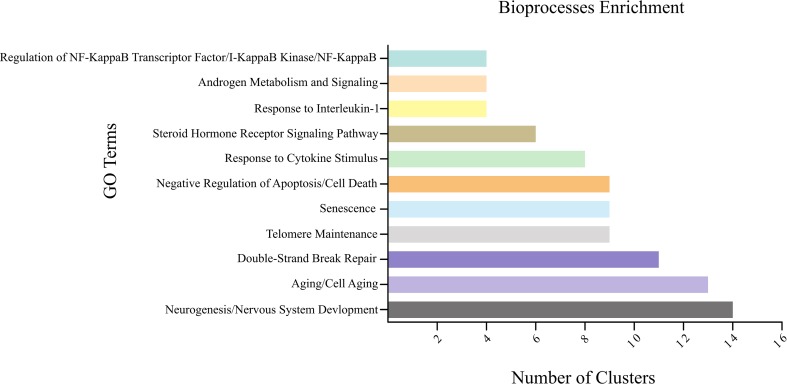
Summary of the bioprocess enrichment identified in the PPI network. The colored horizontal bars show GO terms frequently present in the analyzed clusters.

The most relevant GO terms are listed in Table S1. The main observed terms were: (i) nervous system-associated processes, such as development, synapsis, and learning; (ii) aging; (iii) double-strand break repair; (iv) regulation of apoptosis/cell death; (v) telomere maintenance; (vi) senescence; (vii) response to cytokine stimulus; (viii) regulation of interleukin (IL)-1; (ix) hormone biosynthetic processes, especially androgen biosynthesis; and (x) regulation of the NF-κB/IκB pathway. The relative number of GO terms associated with each cluster can be found in Figure 5. Our analysis excluded GO terms that were not associated with significant biological processes related to the disease, or that were too general (e.g., regulation of biological process, signaling process, or response to endogenous stimulus).

## Discussion

CdCs patients are traditionally diagnosed based on a detailed clinical evaluation and cytogenetic investigations. Furthermore, some studies have shown the importance of characterizing the genomic position of the critical chromosomal region associated with CdCs for a better understanding of genotype–phenotype correlations (Wu *et al.*, 2005; Zhang *et al.*, 2005; Damasceno *et al.*, 2016). Network-based approaches may contribute to the identification of specific genes distributions in a given disease and reveal common molecular mechanisms among genes affected by the condition. Furthermore, genes associated with the same illness have been observed to interact with each other more frequently than expected by chance (Barabási *et al.*, 2011).

### Interaction between SLC6A3 TPPP and CCT5 and Processes related to neuronal development and function in CdCs

In this study, the constructed networks and topological analysis, such as those in clusters 1 and 8 (Figures 2 and 4), showed interactions between SLC6A3, TPPP, and CCT5, genes which are located in the SRO, and interactions between processes related to neuronal development and function in CdCs. The GO analysis of clusters 1 and 8 indicated the presence of proteins deleted in hemizygous individuals in our study that are related to the regulation of glutamatergic and dopaminergic synaptic transmission, catecholamine uptake involved in synaptic transmission, and norepinephrine secretion and neurogenesis. Changes in patterns of neuronal activity modulated by dopamine and noradrenaline in the cortico-striatal region of the brain are able to influence the emergence of disturbances, such as attention deficit hyperactivity disorder (ADHD) (Del Campo *et al.*, 2011; Cummins *et al.*, 2012). Interestingly, ADHD is present in about 70% of children with CdCs (Nguyen *et al.*, 2015), and, in our study, hyperactivity was present in five out of the six subjects (Figure 1). SLC6A3, a dopamine transporter, regulates extracellular dopamine, is responsible for the reuptake of dopamine, and functions to balance levels of neuronal dopamine (Gizer *et al.*, 2009). Deficiency of this protein can lead to the accumulation of dopamine in the cytosol, with deleterious effects (Sotnikova *et al.*, 2005). These effects may be associated with hyperlocomotion, stereotyped behaviors, and hyperactivity, as in *Slc6a3* KO mice (Giros *et al.*, 1996; Pogorelov *et al.*, 2005; Lohr *et al.*, 2017), or decreased immobility, as in *Slc6a3* ^+/–^ mice (Perona *et al.*, 2008). Therefore, SLC6A3 can be proposed as a good target on subsequent functional analyses that could increase the mechanistic knowledge related to those CdCs phenotypes. Interestingly, we observed that TPPP is among the direct neighbors of SLC6A3 in cluster 8 (Figure 4). TPPP functions in tubulin polymerization and microtubule stabilization (Vincze *et al.*, 2006). TPPP plays an important role in pathological conditions through the co-enrichment and co-localization of TPPP and α-synuclein in human brain inclusions, such as in Parkinson’s disease (Oláh and Ovádi, 2014). Through the polymerization of the tubulin polymer, TPPP contributes to the extension of peripheral axons in sensory neurons (Aoki *et al.*, 2014). Changes in the expression of TPPP are associated with the phenotypes of depression and anxiety following early life stress in humans (Montalvo-Ortiz *et al.*, 2016). Therefore, these results identified by network analysis suggest an important perturbation between the proteins SLC6A3 and TPPP generating neural changes in CdCs individuals. SLC6A3 also interacts with the H-B CCT5 in cluster 1, in which processes related to cognition, memory, and learning can be found (Figure 4, Table S2). The protein CCT5 is involved in cilia morphogenesis and neurodegenerative processes, and its deficiency may cause neurodegenerative diseases, such asspastic paraplegia (Bouhouche *et al.*, 2006; Posokhova *et al.*, 2011), supporting the GO results. Individuals with spastic paraplegia may present with atrophy of the spinal cord and defects in the upper limbs. These results indicate that SLC6A3*,* CCT5 and TPPP show important connection. Thus, we could consider that disruption of these interactions may change the processes related to neuronal development and function underlying in some patients with CdCs.

### Interplay between of genes in the SRO and behavioral and cognitive impairment

The proteins encoded by *CTNND2*, *TERT*, and *MED10*, which are located in the SRO determined in this study (Figure 2), are commonly deleted in CdCs and interact in several modules associated with neuronal development/function and cellular death, specifically clusters 3, 5, 6, 8, 10, and 11 (Tables S4, S5, S6, S7, S9, S11 and S12). This suggests an interplay between genes in the SRO and behavioral and cognitive impairment*.* These genes are expressed during important periods of embryonic and neuronal development (Yui *et al.*, 1998; Kwon *et al.*, 1999; Ho *et al.*, 2000). *CTNND2*, considered a bottleneck in our analysis, encodes δ-catenin, a component of adherens junction complexes (Kosik *et al.*, 2005) that regulates spine morphogenesis and synapse function in hippocampal neural cells during development (Arikkath *et al.*, 2009). δ-Catenin is stabilized by N-cadherin, which binds to PDZ domain proteins in the post-synaptic compartment at synapse junctions and regulates spine architecture during hippocampal development and the differentiation of neurons via downstream effectors that bind to actin in the cytoskeleton (Kosik *et al.*, 2005; Yuan *et al.*, 2015). Among the bioprocesses investigated in the protein interaction network, we identified the negative regulation of the Wnt receptor signaling pathway. Through Wnt signaling, δ-catenin prevents Rho GTPase signaling, modulating the Ras superfamily in cytoskeletal reorganization (Lu *et al.*, 2016). Perturbations in this pathway, observed after depletion of δ-catenin, may contribute to functional neurological alterations (Arikkath *et al.*, 2009). In this sense, the loss of a copy of *CTNND2* in CdCs may be associated with intellectual disability, reading problems (Medina *et al.*, 2000; Belcaro *et al.*, 2015; Hofmeister *et al.*, 2015), learning difficulties, and autism spectrum disorder (ASD) (Asadollahi *et al.*, 2014) (Figure 1). The interplay of δ-catenin with cadherin suggests its influence on Wnt/β-catenin signaling (Lu *et al.*, 2016), which increases keloid cell proliferation and inhibits apoptosis through its interaction with telomerase (Yu *et al.*, 2016). This mechanism perhaps explains the enrichment of the negative regulation of apoptosis process in the GO analysis (Figure 5). In addition, reduction in MED10 levels enhances Wnt signaling and is required for the expression of developmentally regulated genes (Kwon *et al.*, 1999; Lin *et al.*, 2007). The H-B MED10 is crucial for DNA-binding factors that activate transcription via RNA polymerase II (Sato *et al.*, 2003). Lastly, the telomerase reverse transcriptase, encoded by *TERT*, which behaved as an H-B, was the most clusterized protein (Tables S15 and S17). The hemizygosity of *TERT* has been associated with shorter telomeres in lymphocytes from CdCs patients and contributes to the phenotypic changes seen in the syndrome (Zhang *et al.*, 2003). However, another study with 52 individuals affected by CdCs showed that the telomere length in CdCs patients was within the normal range, though the average was shorter than that in normal controls (Du *et al.*, 2007). These data suggest that the contribution of *TERT* to CdCs may involve alterations in other biological processes or pathways. For instance, *TERT* can exert protective effects. Under dietary restriction conditions, TERT accumulates in the mouse brain, leading to reductions in free radicals in the mitochondria, DNA damage, and apoptosis through the inhibition of the mTOR cascade (Miwa *et al.*, 2016). These processes were present in all clusters except 1 and 13 (Tables S2 and S14).

Therefore, analyses of centrality suggest that the deficiency in *CTNND2*, *TERT*, and *MED10* genes expression during important stages of development may affect processes related to neurogenesis and the regulation of apoptosis and DNA repair, being inherent in the cognitive and behavioral impairments seen in CdC patients (Figure 1).

### Control of NF-kB transcription factor/interleukin 1 and inflammatory response

In several clusters, GO analysis identified processes related to the immune system and inflammatory response. Considering this, we explored the control of the NF-κB transcription factor/IL-1 and the inflammatory response*.* The appearance of respiratory and intestinal infections during the first years of life is common in patients with CdCs, though it has been rarely discussed (Mainardi, 2006). Processes related to immune response-activating signal transduction, response to IL-1, leukocyte activation, and regulation of the IκB kinase/NF-κB cascade, which has an important role in inflammation (Deacon and Knox 2018), were observed in our study, especially in clusters 1, 2, 3, 5, 6, 7, 8, 9, 10, 11, and 12 (Figure 5, Tables S2 -S4 and S6-S13). With the use of telomerase inhibitors and telomerase-targeting small interfering RNAs, it has been found that H-B TERT reduces TNF-α-induced chemokine expression in airway smooth muscle cells (SMCs) (Deacon and Knox, 2018). Another protein involved in the immune response is adenylyl cyclase (ADCY2), which is also an H-B according to the centrality analysis. This protein catalyzes the formation of cyclic adenosine monophosphate (cAMP) from adenosine triphosphate (ATP), involving various signal transduction pathways. ADCY2 regulates the production of IL-6 in inflammatory processes and enhances its expression in SMCs (Bogard *et al.*, 2014; Jajodia *et al.*, 2016). In addition, single-nucleotide polymorphisms in ADCY2 have been associated with severe chronic obstructive pulmonary disease (Hardin *et al.*, 2012).

These data suggest that the presence of specific pathways related to the immune response can be affected by genes commonly deleted in CdCs (Figure 5). These results bring new insights into the pathogenesis of the syndrome, in an attempt to explain the emergence of recurrent respiratory and intestinal infections during the first years of life in individuals with CdCs (Mainardi, 2006).

### Association between genes in SRO and congenital malformations.

Regarding the association between genes in the SRO and the multiple congenital malformations observed in CdCs, the network analysis demonstrated interactions between MTRR, CEP72, NDUFS6, MRPL36, and MED10 in clusters 2 and 4, in which the GO analysis identified processes related to DNA repair, cell cycle control, cellular death, and mitochondrial ATP synthesis, and electron transport (Figure 5). *MTRR* encodes a methionine synthase reductase that is fundamental for the remethylation of homocysteine, which regenerates functional methionine synthase via reductive methylation. Individuals with neural tube defects (NTDs) exhibit elevated homocysteine concentrations (Steegers-Theunissen *et al.*, 1993; Zhu *et al.*, 2003; Cheng *et al.*, 2015). The protein MTRR emerged as a bottleneck in our protein interaction network. Heterozygous mutations that lead to MTRR deficiency have been implicated in homocysteine accumulation, resulting in adverse reproductive outcomes and congenital heart defects in mice (Zhu *et al.*, 2003; Li *et al.*, 2005). Therefore, defects in the activity of MTRR could be associated with frequent clinical manifestations of CdCs, such as cardiac abnormalities. Furthermore, neurodevelopmental disorders such as primary microcephaly are associated with mutations in proteins that interact with the centrosomes, such as the CEP72 (Kodani *et al.*, 2015), which was considered an H-B in our analysis. CEP72 regulates the localization of centrosomal proteins and bipolar spindle formation (Oshimori *et al.*, 2009). Therefore, CEP72 is involved in centriole duplication and biological processes such as control of the cell cycle, and deficiency of this protein may contribute to dysmorphic phenotypes in CdCs (Figure 1).

Another protein in cluster 2 was the H-B NDUFS6, an accessory subunit of the mitochondrial chain NADH dehydrogenase (Murray *et al.*, 2003). Deletion of NDUFS6 or mutation of its Zn-binding residues blocks a late step in complex I assembly (Kmita *et al.*, 2015). Mutations in this protein may also cause lethal neonatal mitochondrial complex I deficiency (Kirby *et al.*, 2004) and fatal neonatal lactic acidemia (Spiegel *et al.*, 2009). Besides these proteins, MRPL36, a component of the ribosomal subunit (Williams *et al.*, 2004), emerged as a hub in our network of protein interactions. Decreases in MRPL36 prevent the correct folding and assembly of translation products, leading to rapid degradation of these molecules and defects in the biogenesis of respiratory chain complexes in the mitochondria (Prestele *et al.*, 2009). Therefore, the hub MRPL36 may contribute to oxidative stress-related processes found in cluster 2 (Table S3) and may be associated with excess apoptosis and NTDs (Yang *et al.*, 2008).

Excessive apoptosis in fetal central nervous tissues can cause NTDs by decreasing the number of cells in the neural folds or by physical disruption of the dorsal midline, consequently resulting in embryonic dysmorphogenesis (Chen *et al.*, 2017; Lin *et al.*, 2018). Furthermore, the H-B MED10, located in clusters 2 (Figure 4), 3, and 4, regulates heart valve formation in zebrafish (Just *et al.*, 2016). In addition, network analysis demonstrated an interaction between MED10 and the protein encoded by *MED24/TRAP100*, located on chromosome 17. MED24 is necessary for enteric nervous system development in zebrafish (Pietsch, 2006). Together, these findings contribute to our understanding of the emergence of congenital heart defects, microcephaly, and occasional abnormalities such as agenesis of the corpus callosum, cerebral atrophy, and cerebellar hypoplasia, which may be present in CdCs.

## Conclusion

The possibility of using microarrays to characterize chromosomal rearrangements has led to several studies aimed at establishing genotype-phenotype correlations in several contiguous gene deletion syndromes, and some of them have proposed the regions of susceptibility to each specific condition. However, no consensus has been reached on the exact identity of the genes and cell signaling pathways involved in promoting these symptoms, as e.g. in the CdCs. This is the first study to explore the interaction network of the proteins encoded in the critical region associated with CdCs by combining cytogenomic data and systems biology tools. This study identified and demonstrated the biological processes involving genes previously found to be associated with CdCs, such as *TERT*, *SLC6A3,* and *CTDNND2*. Furthermore, through analysis of the protein interaction network, we identified other possible candidate proteins, including CCT5, TPPP, MED10, ADCY2, MTRR, CEP72, NDUFS6, and MRPL36, with potential contributions to the phenotypes observed in CdCs. Further functional analysis of these proteins is required to fully understand their involvement and interplay in CdCs. Additional research in this direction may confirm those that are directly involved in the development of the CdCs phenotype and improve genotype–phenotype correlations.

## Supplementary material

The following online material is available for this article:

Table S1 List of genes located in the smallest overlap region.

Table S2 List of Go terms identified by BiNGO in the Cluster 1.

Table S3 List of Go terms identified by BiNGO in the Cluster 2.

Table S4 List of Go terms identified by BiNGO in the Cluster 3.

Table S5 List of Go terms identified by BiNGO in the Cluster 4.

Table S6 List of Go terms identified by BiNGO in the Cluster 5.

Table S7 List of Go terms identified by BiNGO in the Cluster 6.

Table S8 List of Go terms identified by BiNGO in the Cluster 7.

Table S9 List of Go terms identified by BiNGO in the Cluster 8.

Table S10 List of Go terms identified by BiNGO in the Cluster 9.

Table S11 List of Go terms identified by BiNGO in the Cluster 10.

Table S12 List of Go terms identified by BiNGO in the Cluster 11.

Table S13 List of Go terms identified by BiNGO in the Cluster 12.

Table S14 List of Go terms identified by BiNGO in the Cluster 13.

Table S15 List of Go terms identified by BiNGO in the Cluster 14.

Table S16 List of Go terms identified by BiNGO in the Cluster 15.

Table S17 List of Go terms identified by BiNGO in the Cluster 16.
